# Multilevel implications for anti-consumption social marketing within the public policy framework for SDG realization: a systematic literature review

**DOI:** 10.1007/s12208-023-00375-5

**Published:** 2023-05-29

**Authors:** Olavo Pinto, Beatriz Casais

**Affiliations:** 1 School of Economics and Management, University of Minho, Campus de Gualtar, 4700-057 Braga, Portugal; 2 CICS.NOVA. UMinho, University of Minho, Braga, Portugal

**Keywords:** Anti-consumption, Public policy, Social marketing, Sustainability, Systematic literature review

## Abstract

This systematic literature review analyzes the topic of anti-consumption within the framework of public policy and discusses the multilevel implications for social marketing. Previous research provides a broader scope of analysis based on cases suggesting public policy implications of anti-consumption. However, the topic broadens into social issues and calls for the discussion of the social role and the relationship to sustainability. Building on the goal number 12—Responsible Consumption and Production—of the United Nations Sustainable Development Goals (SDGs), the authors analyzed 42 peer-reviewed papers to assess the relationship quantitatively and qualitatively between public policy and anti-consumption, pointing to future avenues of research. The results show how policymakers can address either disruptive or transitional approaches by considering systemic changes. Public infrastructure and public management are important factors to support policies aiming to achieve sustainable and replacement consumption. However, participatory and transparency mechanisms are needed to effect this social change, which reaffirms the importance of stakeholders and the analysis of their relationships. The impact of anti-consumption on macro and structural changes may be hard to measure, but should not be dismissed. This paper calls for a broad approach to anti-consumption and the mapping of stakeholders – including individuals, organizations, governments, researchers and the media – by applying a social marketing perspective to sustainability concerns. By linking anti-consumption both with social marketing and the contemporary challenge of environmental sustainability reflected on the SDGs, this paper bridges the gap between individual analysis of anti-consumption and its impact and potential to address sustainability challenges.

## Introduction

Anti-consumption as a practice or lifestyle has been generating increasing interest in management, business, and economic literature (Makri et al., 2020) as a set of actions by consumers or brands that, within a group environment, result in the choice to abstain from consuming, consume less, or change consumption patterns to accommodate anti-consumption goals (Albinsson & Yasanthi Perera, 2012; Cherrier, 2009). A large number of these anti-consumption manifestations occur deliberately within the framework of marketing system of meaning. As such, they are expressed within a controlled or close-brand context level, emerging in the form of brand relationships (Fetscherin, 2019; Fetscherin & Kc, 2021; Hegner et al., 2017; Yuksel & Mryteza, 2009; Zarantonello, 2020). The approach to anti-consumption has provided several conceptualizations of how consumers, brands, and other stakeholders engage in these types of actions (Iyer & Muncy, 2009). Anti-consumption can be applied to a broader context that expands from the current micro level of analysis. While this type of analysis provides insightful and relevant perspectives, there is a call in the literature to open the context of this analysis toward society and relevant stakeholders, considering global challenges facing society, such as sustainability and environment (Kuanr et al., 2021; Seegebarth et al., 2016).

Anti-consumption is a growing concept in consumer behavior, marketing, and psychology regarding consumption and lifestyle phenomena. However, while it is an underlying mechanism for lifestyle, health, and sustainable development studies, its contributions lack a solid connection to many of the problems it decries. While its focus is usually on individual consumer behavior, little attention has been paid to the public policy implications derived from the individual orientations, although their importance is recognized (Nepomuceno & Laroche, 2017; Piacentini & Banister, 2009). By contrasting existing knowledge and topics of interest in the anti-consumption arena that are relevant to public policy, this research contributes to broadening and provide an overlap of anti-consumption, sustainability, and social marketing.

This study highlights the opportunities that arise in the anti-consumption literature to address sustainable and environmental challenges through social marketing, especially upstream social marketing. This systematic literature review is relevant and innovative, as it stems from current knowledge on anti-consumption, and aims to contribute to an approach with broader frames of analysis to integrate the discussion of relevant social issues, such as the climate crisis (Lee & Ahn, 2016). Public policy has implications characterized in social marketing and their contribution to further research on anti-consumption can clarify mechanisms of change and pursuit of well-being (Domegan et al., 2019; Gordon, 2013; Issock et al., 2020; Lee & Ahn, 2016) or social good (Wymer, 2021). Social marketing has helped address issues related to health and environmental sustainability and some of the sustainable development goals (SDGs) identify anti-consumption and policy implications. Although not often discussed within extant anti-consumption literature, it broadens the perspective on anti-consumption (Hall, 2016).

The COVID-19 pandemic provided important examples of how social marketing can be helpful to address social, public, and mental health issues (Akdas & Cismaru, 2021). The pandemic disrupted the economic and marketing systems and impacted the individual well-being, highlighting anti-consumption approaches and perspectives. The invasion of Ukrainian territory by Russia is also causing a disruption in the global market and has brought to the forefront the complex system of international relations, in which anti-consumption policies, negotiated through demands and sanctions, interact with the perceptions of a global crisis.

Anti-consumption proposes relevant avenues for a critical framework for consumption and marketing systems as root causes of anti-consumption in society (Gordon, 2011). Social marketing can be useful addressing complex problems in multi-environmental contexts, as well as public policy impacts and relationships. Further, sustainability policies and goals require the involvement of stakeholders and a holistic perspective over media, but also companies and individuals, employees, consumers and researchers (Wymer, 2021). Responsible consumption and production correspond to UN SDG number 12 and reflects the desired path of societies towards a common good. These governmental agendas resonate with public policy and impact both the economic system and future societies. Social marketing is relevant for anti-consumption, as it addresses interesting issues raised in the literature (Lee et al., 2013; Wymer, 2021) and distinguishes immediate responses to solve it as it unfolds (downstream intervention), from engendering social action aimed at preventing and addressing the root causes of an issue (upstream intervention). However, considering upstream intervention, public policy can be useful in achieving structural and effective change more than to implementing immediate action (Hall, 2016). While the United Nations and other supranational organizations are governed by a holistic approach, it is essential to study all relevant stakeholders to address global social issues (Domegan et al., 2019). In this sense, this study proposes a relevant link between public policy and anti-consumption to provide a valuable alignment between global issues, the contribution of literature in addressing anti-consumption phenomena, and the context of social marketing, where consumer behavior and consumer culture merge.

## Conceptual background

### Anti-consumption

Anti-consumption refers to the behavior or intention to “reject, reduce or reclaim certain goods, services, or brands” (Lee & Ahn, 2016, p. 18), and may lead to outright rejection, avoidance or cessation. Authors such as Lee et al. (2011) and Paschen et al. (2020) differentiate anti-consumption from both consumer resistance and intentional behavior. Anti-consumption can manifest as avoidance, resentment (Zavestoski, 2002), rejection, restriction, and reclamation of a good or service (Lee & Ahn, 2016). Different categories have thus emerged in the consumer decision process, whether they are political, social, or ideological in nature (Lee et al., 2011). Anti-consumption can target specific brands, consumption patterns or attitudes, and countries of origin of particular products. Consumer resistance emerges as an opposing force to the market system (Cherrier, 2009), whether via boycotting (Kozinets & Handelman, 1998) or alternative market consumption practices, which are two of the most extreme examples of anti-consumption (Albinsson & Yasanthi Perera, 2012; Lee et al., 2011).

The consumer decision process encompasses anti-consumption problematization that is manifested in different cultures and different socioeconomic segments of the world’s population (Gossen et al., 2019). Anti-consumption behaviors are narrowly presented in the literature and lack generalizability (Dholakia et al., 2018; Ganassali & Matysiewicz, 2021). Some studies on the anti-consumption phenomenon depend on groups (Albinsson & Yasanthi Perera, 2012; Kuanr et al., 2020) and lack a comprehensive and generalizable conceptualization (Fernandez et al., 2011). However, new studies on broader anti-consumption applications, like environmental sustainability orientation, can shed light on a broader scope in which anti-consumption is applied not only to consumers, but also to individuals, citizens, and other stakeholders in the production, regulation, and leadership roles (Defila & Giulio, 2020; Paschen et al., 2020; Ziesemer et al., 2021).

Anti-consumption is often related to over-consumption, since it is a rejection of consumerism, a reaction of a society that seeks to restore the balance of forces within capitalism (Chatzidakis & Lee, 2013) by criticizing its methods of excessive waste, ineffective innovation, logistic or environmental practices. In many cases, the reaction to consumption is often reflected in new markets, new segments, and new target audiences for products and services, created from the destruction of existing market systems. This reaction emerges as an innovation and creative driver that stems from the system’s own internal conflicts, as well as from the consumer’s conflicts against consumption, or from the producer or the market that question their own operationalization and aims to find solutions to their needs. Like creative destruction, an expression coined in the mid-twentieth century by Schumpeter (2019) to describe how capitalist systems replace each other due to their operating model, anti-consumption also analyzes societal innovations regarding consumption and market systems, replacing consumption with innovative solutions, trends and behavioral expressions of ownership that satisfy needs in individual or collective perspectives. At the same time, the drivers of combating consumption are not the mere opposite of consumption (Chatzidakis & Lee, 2013). Consumption holds an important symbolic meaning for the consumer and his environment (Hogg et al., 2009; Sussan et al., 2012). Current literature is not consensual about the limits and implications of anti-consumption, but novel applications can broaden its focus, such as the case of the historical analysis of prohibition by Witkowski (2020) and the newly coined environmentally oriented anti-consumption to find sustainable implications from anti-consumption (García-de-Frutos et al., 2018). Thus, anti-consumption does not restrict its focus to consumer behavior, and social issues such as the sustainable future demand that it be framed in a social questioning that involves multiple stakeholders in the consumption system.

### Social marketing for anti-consumption

Current literature on consumer behavior has focused on individual anti-consumption responses such as brand hate, brand avoidance or cessation (Cherrier, 2009; Kucuk, 2008; Lee et al., 2009; Varman & Belk, 2009). This study allows for expansion of knowledge on anti-consumption and demonstrates how the literature has been providing public policy with valuable insights on social issues in which anti-consumption may prove useful. Social marketing focuses on behavioral changes needed to improve structures, processes or interactions in the social system. But it also focuses on strategic goals, and in that sense upstream social marketing can contribute to evidence-based public policy (Gordon, 2013). While downstream social marketing focuses on the immediate, individual action on a given social issue, this paper focuses predominantly on the application of upstream social marketing to issues where anti-consumption concerns, and its relationship with public policy.

Social marketing research urges to uncover how the individual faces negative consequences, and it can positively contribute to critical and operational recognition within a societal dynamic. Social marketing aims for behavioral change in individuals (Gordon, 2013; Hall, 2016; Wymer, 2021). However, according to the upstream perspective of social marketing, there are many other considerations beyond the individual. In this sense, it seeks to implement, discuss and develop the mechanisms and practices of public policy with a view to achieve social change (Gordon, 2013). Research in this area requires a transparent and participatory approach to finding solutions, rather than top-down policies, and multiple strategic and contextual considerations are indispensable (Domegan et al., 2019; Gordon, 2011, 2013; Mehmet & Simmons, 2019).

The contribution of these marketing tools to structural and sustainable change must be properly assessed to ensure that it does not merely contribute to their own declared goals (Gossen et al., 2019). New marketing solutions to environmental sustainability problems, as well as the role of the consumers in distinguishing, selecting, and making that choice to change, are subject to how public policy is implemented – whether through education programs, labels or policies). The effect of consumer behavior on environmental sustainability in a global marketplace assumes all the relationships between these actors (Arnould, 2007). Different cultural values and different societal norms present different approaches to consumption, and this also applies to green consumption (Kuanr et al., 2021). How brands use anti-consumption contributes to their performance. These approaches can bring benefits for the consumer, the producer, and society at large, even if circumscribed in time and scope (Sekhon & Armstrong Soule, 2020).

As social marketing activates anti-consumption mechanisms, public policy moderates its action, and is present in education programs, tax, regulatory and restriction measures (Evers et al., 2018), as well in health and wellness programs (Fry, 2010; Piacentini & Banister, 2009). Social marketing understands the importance of marketing in achieving change, and its applications rely on both the marketing mix and a critical examination of marketing systems. Social marketing advocates a dynamic relationship between stakeholders while at the same time analyzing and providing information on social phenomena such the problem of environmental sustainability and the role of anti-consumption, given that its purpose is to achieve the well-being of individuals within their societies (Gordon, 2013; Gossen et al., 2019). The United Nations consider sustainable consumption and production to be a crucial change (United Nations, n.d.), and these goals are in line with many of the public initiatives related to anti-consumption practices (Ziesemer et al., 2021). The stakeholders most responsible for setting and assessing goals are governments and agents responsible for policymaking (Gossen et al., 2019; Isenhour, 2010; Ziesemer et al., 2019) and their implementation (Ganassali & Matysiewicz, 2021; Lee et al., 2018).

Public policies do not exclusively impact consumption, but also product development, transportation, production and storage (Martin et al., 2019) that are more directly affected. Public policy and environmental sustainability impact the regulation of products and processes with environmental implications (Linton et al., 2007), and different parts of the supply chain contribute to a more – or less – sustainable production system (Denac et al., 2018; Martin et al., 2019). The role of public policy and regulation is essential for the development of governance among commercial institutions, despite differences in sectors, contexts, and common practices by country (Boons et al., 2012; Gordon, 2013). From a critical perspective, marketing is a pillar of the system, in which consumption, considered as a comprehensive vector of change for social good, can benefit from anti-consumption problematizations (Gossen et al., 2019). It is through anti-consumption that many environmental sustainability-oriented policies realize their potential, and the social marketing approach to these social issues raises an interesting connection in the literature.

### Sustainability and anti-consumption

According to the UN, the major problems to be tackled by the development programs relating to SDG number 12, Responsible Consumption and Production, created to measure, promote and accelerate the adoption of sustainable practices from the major stakeholders are inefficient resource use, waste disposal, underreporting of sustainable practices, and inefficient and polluting energy use (United Nations, n.d.). The anti-consumption literature has presented extensive research related to the negative consequences of anti-consumption, from the perspective of brand-controlled interactions (Fetscherin & Kc, 2021). However, anti-consumption encourages more sustainable and responsible consumption with environmental benefits, but also contributes to a wider performance of sustainability in general, which can benefit from anti-consumption not only in its environmental dimensions, but also with social and institutional manifestations.

Directly aligned with UN’s SDG number 12, anti-consumption helps to understand, both at the individual and institutional level, which consumption practices are useful in creating more sustainable and responsible consumption patterns to promote a more sustainable future. Anti-consumption encourages sustainable consumption practices, since it promotes a reduction in consumption activities, thus rejecting and minimizing unnecessary consumption and some of its direct impacts on emissions, waste and on individual and social behaviors. Buying less, reusing, and repairing are practices that exploit fewer resources and generate less harmful emissions. For producers, anti-consumption goals can motivate them to follow innovation requirements from a consumer-oriented perspective to improve the efficiency of the production chain and thus meet these requirements. Institutions have been striving to meet normative requirements to achieve sustainable goals and marketing activities that support and contribute to the creation of a sustainable society.

Using institutional and governmental action to achieve these goals, anti-consumption is indirectly guided by climate action and sustainable resource use goals, as defined in UN SDG number 13 (Take urgent action to combat climate change and its impacts) by reducing emissions and waste generation, which are a direct consequence of high levels of consumption and production. Climate change is a direct effect of human action, and consumption is responsible for a large share of greenhouse emissions (González et al., 2011; O’Shea et al., 2013). Resources, transportation, and energy generation are impacted by consumption, and the transition to renewable energy may exacerbate the burden of consumption on environmental sustainability. The exact impact of anti-consumption on environmental sustainability is unknown, but this link is theoretically implied (Nepomuceno & Laroche, 2017). Other challenges addressed by the UN are SDG number 8, sustainable tourism, and especially SDG number 3, good health and well-being, which are a studied consequence of anti-consumption practices, both at the individual or group level, such as voluntarily opting for a simpler life. These challenges are addressed by social marketing research and are studied in their implications for consumption and public policy (Akdas & Cismaru, 2021; Domegan et al., 2019; Hall, 2016; Wymer, 2021). Individual well-being merges individual good and common good in a complex interrelationship of both practices and public policies increasingly focused on the efficient use of resource and its impact on the final consumer (Black, 2010; García-de-Frutos et al., 2018; Ziesemer et al., 2021). The effective use of resources also contributes to the preservation of ecosystems, as stated in SDG numbers 14 and 15, by finding alternatives to the high volumes of resource exploitation of oceanic and terrestrial ecosystems and consequent effects such as deforestation and threats to endangered species (Linton et al., 2007; Scott & Weaver, 2018). Effective resource use can be directly affected by anti-consumption since it helps to set a climate and resource use threshold. Water, raw materials, land use, and mining exploit scarce resources and greatly impact present and future generations.

Consumer choice can also have a sustainable impact by supporting labels or brands characterized by less harmful, sustainable or green production methods in the industry, or by social and economic justice in terms of the labor involved in production (Rajagopal et al., 2017). Social justice is indirectly affected by anti-consumption, through consumer demand, and especially impacts the perception norms and practices considered unfair or outright wrong in terms of labor and the environment (Ortega-Egea & García-de-Frutos, 2021). From an anti-consumption perspective, not all consumption has equal weight, and the sustainable, welfare or retaliation models described in the anti-consumption literature ultimately seek a system change, sometimes with a global social and natural impact. To this end, the individual or the institution may use different approaches in managing this change within the consumption system.

There is in many contexts a systemic social issue inherent to anti-consumption that focuses especially on the relationship between sustainability and policy. Social marketing can be instrumental in driving change (Gossen et al., 2019), and the influence of policy is important in complexifying individual behavior change (Domegan et al., 2019). Anti-consumption practices, instruments, and policies are paramount in developing both consumer and institutional strategies and mechanisms to address social issues. The dynamic transformation of markets through policy and interaction of actors does not yet integrate anti-consumption as a sustainable concept.

### Anti-consumption, public policies, and relevant stakeholders

Since consumption implies a choice, anti-consumption draws a line where all alternatives are purposefully and intentionally not chosen (Lee et al., 2018). Symbolic meaning affects consumer choice, and is strongly associated with environmental sustainability motivations (Kropfeld et al., 2018; Scheurenbrand et al., 2018; Wooliscroft et al., 2014), health and wellbeing (Fry, 2010; McArthur, 2015; Piacentini & Banister, 2009; Yarimoglu et al., 2019), or other positive outcomes associated with cessation or abstention from consumption. This reduction, avoidance, and cessation are choices made in the market system and are individual anti-consumption behaviors as mechanisms to solve social problems to achieve sustainable goals. In order to understand and achieve a systemic goal and public policy (Gordon, 2013) social marketing has focused on what constitutes our social reality and what can change it (Domegan et al., 2016).

Public policies play a role in determining both individual and institutional activities. Some examples are regulation and taxation used with the aim of lower consumption, with implication in analyzing countries (Gao, 2018) and wider policies, like the plastic bag levy studied as an anti-consumption mechanism used by public policy to promote environmental sustainability (Zen et al., 2013). Taxation and subsidization are some of the economic incentives that allow policies to change the way individuals and institutions use, produce and interact with certain goods and services. Societies promote the adoption of innovations or practices that contribute to the social good while burdening those that have a negative impact, thus promoting a mechanism of change among different actors (Gordon, 2013). However, by examining and developing knowledge on anti-consumption, governments and researchers can determine, in a complex system, more effective and efficient anti-consumption public policies. Other policy mechanisms contemplated in the literature relate to education and wider dissemination programs, contributing to consumer choice in anti-consumption settings, with examples focused on nutrition (Allen et al., 2018; Yarimoglu et al., 2019). The effectiveness of these mechanisms in promoting sustainable practices have been discussed (Lasarov et al., 2019). Educational or dissemination activities, usually funded and driven by governmental programs, are generally intended to educate the public about a social practice that aims to positively impact society. This is the case with recycling or water use reduction education programs, which are permeated by public policies that impact education and communication awareness programs for the public (Evers et al., 2018).

Public policy programs that include educational efforts are one of the mechanisms towards which research tries to contribute (Chatzidakis & Shaw, 2018), however not followed with a direct study of its implications or implementations. Education has, however, been considered as an important part to actively pursue public policy anti-consumption in the case of environmental sustainability (Ziesemer et al., 2021). Messages promoted by public policy are used in efforts to promote direct anti-consumption, for example in the drinking culture (Piacentini & Banister, 2009).

Regulatory measures have direct and indirect impacts on anti-consumption practices through measures such as prohibition, rationing, education or dissemination and through pricing with examples on different levels of the production chain or on final consumers. Therefore, governments can use public policies as a tool that promotes anti-consumption and sustainable goals (Fry, 2010; Linton et al., 2007). Collaborative consumption, which can share some of the anti-consumption environmental sustainable aims, calls for some governance and regulation questions to face it effective implementation and attractiveness to a larger set of consumers (Hartl et al., 2016) and public policy mechanisms of introduction of these policies drive interest for further research in the literature (Gollnhofer, 2017). The banning of chemicals, some of which cause greater climate change through emissions, is a call for social marketing oriented by sustainable goals such as carbon reduction (Gordon, 2011). Anti-consumption research has been related to social policy problems such as waste and obsolescence (Anderson et al., 2018; Guillard, 2018; Hartl et al., 2016; Lee et al., 2018), and public investment in reuse, recycling, and other social programs constitutes a body of regulations that use anti-consumption practices to achieve sustainable goals.

**Fig. 1:**
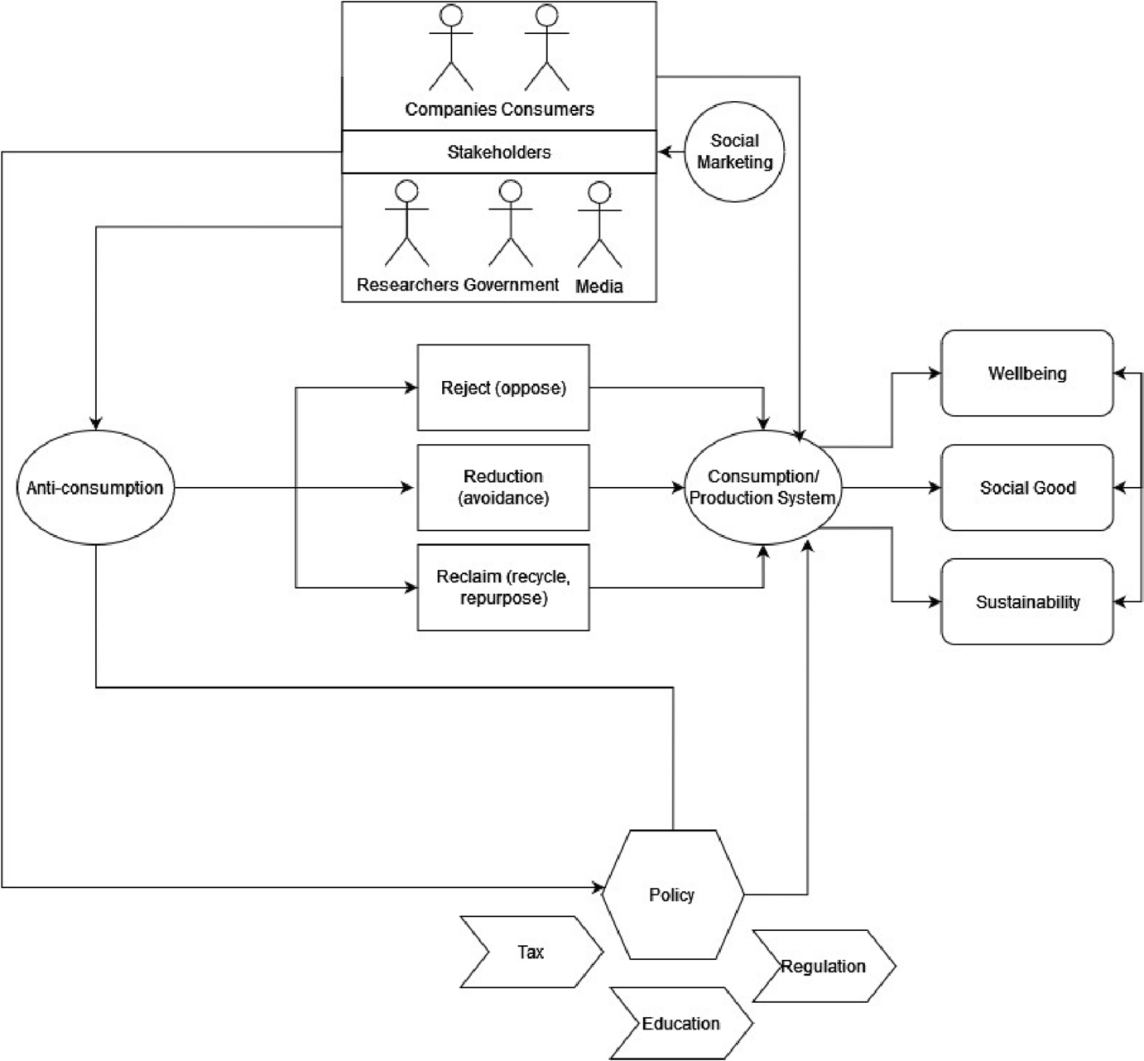
Relationship of anti-consumption with stakeholders and public policy. Source: own elaboration

Beyond wellbeing (Lee & Ahn, 2016) or individual ideology (Cherrier, 2009), anti-consumption works as a mechanism of change within a system that faces a global threat. Anti-consumption places the responsibility on public institutions and stakeholders relationships – a problem not only extensively described in the upstream social marketing literature (Gordon, 2013), but also the subject of critical social marketing analysis (Gordon, 2011), and systems thinking (Domegan et al., 2016). Cohesion in determining relevant stakeholders is crucial to achieving global holistic goals, such as those proposed by the UN SDGs (Domegan et al., 2019). The phenomenon of anti-consumption can affect not only consumers, but also brands, countries, or ideas of choice in the search for positive outcomes for consumers, society, or companies, with brand aversion or overconsumption being illustrative examples of this research (Chatzidakis & Lee, 2013; Hutter & Hoffmann, 2013). Most stakeholders are consumers and citizens directly and indirectly affected by such public policies and social issues. As consumers their needs should be met efficiently while preserving the social good, with the key goal of contributing to their wellbeing. Stakeholders are generally identifiable in public, more specifically as activists or promoters (Ziesemer et al., 2021). When relating to policy, governments, politicians and public administrators, these stakeholders have the responsibility to develop and implement policies that often relate to or can benefit from anti-consumption practices. At the same time, these actors benefit from research on sustainability and anti-consumption to make efficient and beneficial decisions. Companies can use anti-consumption opportunities to accelerate innovation and promote new marketing approaches, even though reducing consumption runs counter to their economic orientation. These actors are at the center of the market as producers of goods users of resources, but they have also been playing an increasing role in the environmental sustainability of these economic uses. Their social responsibility towards consumers and the workforce makes them a cornerstone for public policy to realize its objectives. The media is critically essential for the discussion, dissemination and understanding of anti-consumption and social issues, which can be translated into sustainable practices that promote social good and manage the prioritization of these issues on the political and social agenda. Researchers are responsible for providing valuable information for policymaking based on efficient and correct decisions on policymaking, on the perception and response and among consumers and companies. Figure 1 presents a model of the framework developed by these research advances, which connects upstream social marketing with anti-consumption.

The examples of upstream and downstream social marketing apply to the criticism of overconsumption and fit with many of the consequences and drivers of anti-consumption (Gordon, 2013). Social marketing’s appeals to a broader perspective can help to anti-consumption, as they overlap with individuals, companies, and governments (Gordon, 2013). Multi-level marketing research (micro, meso, and macro) makes it possible to bridge the gap between the individual and the institutional level (Hutter & Hoffmann, 2013), and this can be further sustained with knowledge developed on anti-consumption. The anti-consumption literature makes recommendations for policymaking, and the gap between research results and managerial effectiveness is noticeable, especially on the issue of policymaking, an issue also found in the social marketing literature (Gordon, 2013). The management literature have given great attention to the assessment of food lifecycle (Benedetto et al., 2014; Brodt et al., 2013; Cerutti et al., 2016; Tukker et al., 2011; Vázquez-Rowe et al., 2013), energy and construction industries (Giurco et al., 2011; Schulz, 2010; Zhang & Colosi, 2013), showing that the impact can be mitigated by measuring and analyzing these results. However, social wellbeing and individual wellbeing are not limited to sustainability and efficiency gains along the production chain, but constitute a social challenge in themselves (Domegan et al., 2019; Gordon, 2013; Issock et al., 2020; Lee & Ahn, 2016; Wymer, 2021). To develop a better understanding of contemporary challenges to society, management, and market system that thrive on recent and emerging trends, there is a need to focus on research constraints, stakeholder analysis and policymaking (Chatzidakis & Lee, 2013). Environmental sustainability is a global manifestation of individual and governmental calls for policy development (Liu et al., 2020). The concept of anti-consumption can realize its usefulness in achieving social goals, through the understanding and operationalization both of practices and policies to support sustainable goals for brands, consumers and governments that are oriented towards anti-consumption.

## Methodology

For this research paper, the authors conducted a systematic literature review to gain a full perspective on anti-consumption and policy implications, either from studies providing a thorough account of the interaction of these concepts or those that aim to inform policymakers or policy regulators with valuable considerations.

This literature review only uses highly rated databases such as peer-reviewed articles in international journals. Our study used the search string "anti-consumption” or "anticonsumption" or "anti consumption", “policy” or “policies”, and “regulatory” or “regulation” giving found 42 peer-review articles up to 2022, on Scopus and Web of Science (WoS). We analyzed both empirical and theoretical studies in the categories of Business, Economics, Management and Psychology. Book chapters, books, conference proceedings, and anything other than peer-reviewed articles or early-access peer-reviewed articles were excluded. The results obtained were merged into one database, using WoS in the case of duplicate items. The selected authors are shown in Table 1.

**Table 1:** Search criteria

Criterion	Inclusion		Exclusion
Language	English		Any other than English
Type	Peer-reviewed articles; Early Access of Peer-reviewed articles		Book chapters, Books, Conference proceedings
Study type	Empirical or Theoretical		
Date	Not restricted		Not restricted
Relevance	Anything that analyzed anti-consumption or voluntary consumption reduction, including public policy, healthcare		Not restricted
Categories	Business, Economics, Management, and Psychology		Any other
Databases	Web of Science (WoS), Scopus		
Search string			
"anti-consumption” or "anticonsumption" or "anti consumption" and “policy” or “policies” and “regulatory” or “regulation”			
Total output		42 papers	

A multi-step analysis was performed to conduct this systematic literature review, starting with the reading of titles, keywords, abstract, and sources, which provided a first categorization of topics, context, and, in some cases, methodologies and concepts used. Further reading of abstracts and research papers provided a clearer overview of these categorizations that were corrected. The final analysis of this systematic literature review provided a deeper understanding and clear description of the field. This process is considered good systematic literature review practice (Moher et al., 2009). The total number of publications considered in this study was 42, and no publication was excluded for failing to meet the defined criteria. Table 2 presents a list of the ten most important journals with publications in the field, sorted by the number of publications, with a brief description of the journals and their articles.

**Table 2:** Top 10 journals by number of Articles

Journals	No. articles
*Journal of Public Policy & Marketing*	8
*Journal of Business Research*	5
*British Food Journal*	3
*Journal of Macromarketing*	3
*Journal of Consumer Behavior*	2
*Journal of Business Ethics*	2
*Environment, Development and Sustainability*	1
*Consumption Markets & Culture*	1
*European Journal of Marketing*	1
*Journal of Marketing Management*	1

The *Journal of Business Research*, the *Journal of Public Policy & Marketing*, and the *Journal of Consumer Behavior* are the most cited, followed by the *British Food Journal*. Also worth mentioning are the *Environment, Development and Sustainability*, *Consumption Markets & Culture*, and the *European Journal of Marketing*, all three with only one publication in this field, but which yield more than 40 unique citations each.

**Fig. 2:**
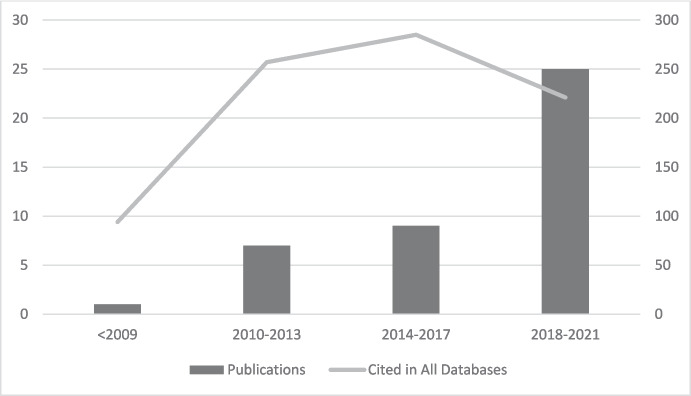
Distribution of publications and citations in 2009–2021

As shown in Fig. 2, the total number of citations for the topic has attracted greater attention over the last decade, with a huge increase in the number of publications on the subject.

## Results

Based on the literature review, most publications (29) shed light on public policy implications, but only 12 of them include the concept of policymaking in their research analyses, either by considering data or information from policymakers or by considering public policies as object of analysis or in correlation with new research implications. Thematic analysis provided insights into the thematic components of the production under study. Most of the research concerns multiple forms of sustainability and health. Many of the publications focus on how alcohol or food can be rejected/reduced to achieve healthier and positive outcomes, especially for young people (Farah & Shahzad, 2020; Khan et al., 2019; Piacentini & Banister, 2009; Yarimoglu et al., 2019). All health topics are categorized under the domain of Health. Sustainability factors that arise from environmental problems, related either to production or consumption and the impact of human activity in the environment, are categorized under Environmental Sustainability. Consumer lifestyle is the third most investigated topic, where voluntary simplicity or variables associated with reduced consumption can impact consumer identity both at the individual or group level (Kropfeld et al., 2018; Lee et al., 2018). Lifestyle factors, such as voluntary simplicity or collaborative markets, were categorized as Lifestyle distinguishing them from consumers' individual choices within the marketing system, that were categorized under Consumer Behavior. It is also worth mentioning that lifestyle-themed articles present plenty environmental sustainability or health considerations, but their focus on other antecedents may not be an intentional part of the individual’s activity. Table 3 describes the frequency of each of the coded topics that are found in the literature review.

**Table 3:** Frequency of publications by main topic

Topic	Count
Health	11
Environmental sustainability	13
Lifestyle	12
Consumer behavior	6

The most used methodology under analysis is the Survey, followed by Ethnography (and netnography) and Interviews, as per Table 4. Ethnography often includes Interview methods, but its application follows other relevant ethnography methods. Discourse Analysis is based on the use of media or marketing collateral.

**Table 4:** Frequency of publications by used methodology

Methodology	Count
Survey	22
Ethnography	8
Interview	7
Discourse analysis	2
Secondary data	2
Theoretical	1

Contributions to the anti-consumption conceptual framework revolve around a dynamic set of interactions between agents by considering all stakeholders in the system (Ganassali & Matysiewicz, 2021; Hogg et al., 2009; Lee & Ahn, 2016). By considering an individual’s social, economic, environmental, political, and legal aspects that can impact changes in consumption patterns, the relationship between anti-consumption and policymaking can broaden the theoretical basis of anti-consumption by taking into account their dynamic interaction in different cultures (Kuanr et al., 2020). Frameworks such as those presented by Gossen et al. (2019) on anti-consumption, marketing, and public policy (Lee et al., 2018) complement future paths that can assess new, underexplored questions. The orientation of social marketing towards action applied to explicit issues reaffirms the theoretical support for the empirical testing of anti-consumption. As such, social marketing orientation needs to be grounded in a scientific process that aims to explain, reproduce, and generate a deeper understanding of a contextual phenomenon and help grow the knowledge base for different applications (Levit & Cismaru, 2020). Although much of the research focuses on general anti-consumption products or activities, some of them are applied to specific activities, where Table 5 provides the frequency of articles within these categories.

**Table 5:** Research product or activity focus

Product/Activity	Count
General	18
Alcohol	4
Food	4
Boycott	3
Repurpose	3
Alternative markets	2
Country of origin	2
Automobile	1
Cycling	1
Plastic tax	1
Social welfare	1
Travel	1
Vaccine	1

When considering Environmental Sustainability, the approaches of the articles under study have a General focus with examples from Isenhour (2010), Kropfeld et al. (2018) and Lasarov (2019) with some applications to specific countries. With a specialised activity under Environmental Sustainability there is Automobile from Wiedmann et al. (2011), Plastic Tax, which deals with the plastic bag policy by Zen et al. (2013), and Travel by Culiberg et al. (2022).

In the Health categories, Alcohol consumption culture is one of the main focus of research for health, with examples of both policy and social marketing activities regarding this social issue with articles from Fry (2010) and Piacentini and Banister (2009). Food is considered within Health with applied research to organic food and issues related to health impact on consumers nutrition (Allen et al., 2018; Ashraf et al., 2019; Farah & Shahzad, 2020). Finally, vaccination appears one time (Demirbag Kaplan & Cem Kaplan, 2011).

Within Consumer Behavior studies, Boycott figures as one of the main themes (Bret Leary et al., 2019; Hoffmann et al., 2018; Yuksel & Mryteza, 2009) but also Country-of-Origin (García-de-Frutos & Ortega-Egea, 2015; Ortega-Egea & García-de-Frutos, 2021) and Alternative Markets (Hartl et al., 2016) figure as relevant topics. When considering Lifestyle themes, there is more focus on General activities. Based on ethnography and interviews, they have specific examples of Repurpose (Anderson et al., 2018; Guillard, 2018; Scott & Weaver, 2018), Cycling (Scheurenbrand et al., 2018) and Social Welfare (Cherrier & Hill, 2018).

However, there are examples in the literature of anti-consumption that combine demographic (Nepomuceno & Laroche, 2015), individual (Kropfeld et al., 2018; Zen et al., 2013), and cultural analysis (Gao, 2018; Isenhour, 2010), contributing to assess how different anti-consumption applications can contribute to policy-oriented knowledge. Illustrating the current state of the literature, Table 6 displays articles with 10 or more citations according to their theme, method and product or activity object of study.

**Table 6:** Articles with 10 or more citations, ranked by number of citations, including the selected theme, method and product/activity object of study

Authors (Year)	Article title	Theme	Method	Product/ Activity	Times cited
Hartl et al. (2016)	Do we need rules for what's mine is yours? Governance in collaborative consumption communities	Consumer Behavior	Survey	Alternative Markets	108
Piacentini and Banister (2009)	Managing anti-consumption in an excessive drinking culture	Health	Qualitative	Alcohol	94
Isenhour (2010)	On conflicted Swedish consumers, the effort to stop shopping and neoliberal environmental governance	Environmental Sustainability	Ethnography	General	71
Zen et al. (2013)	No plastic bag campaign day in Malaysia and the policy implication	Environmental Sustainability	Interviews	Plastic Tax	53
Wiedmann et al. (2011)	Adoption barriers and resistance to sustainable solutions in the automotive sector	Environmental Sustainability	Survey	Automobile	47
McArthur (2015)	Many-to-many exchange without money: why people share their resources	Consumer Behavior	Ethnography	Alternative Markets	45
Nepomuceno and Laroche (2015)	The impact of materialism and anti-consumption lifestyles on personal debt and account balances	Lifestyle	Survey	General	43
Fernandez et al. (2011)	Doing the duck: negotiating the resistant-consumer identity	Lifestyle	Ethnography	General	42
Kropfeld et al. (2018)	The Ecological Impact of Anticonsumption Lifestyles and Environmental Concern	Environmental Sustainability	Survey	General	30
Nepomuceno and Laroche (2017)	When Materialists Intend to Resist Consumption: The Moderating Role of Self-Control and Long-Term Orientation	Lifestyle	Survey	General	30
Fry (2010)	Countering consumption in a culture of intoxication	Health	Interviews	Alcohol	28
Ashraf et al. (2019)	Consumers’ anti-consumption behavior toward organic food purchase: an analysis using SEM	Health	Survey	Food	26
Wooliscroft et al. (2014)	The Hierarchy of Ethical Consumption Behavior: The Case of New Zealand	Environmental Sustainability	Survey	General	20
Hackley et al. (2015)	Transgressive drinking practices and the subversion of proscriptive alcohol policy messages	Health	Qualitative	Alcohol	16
Hoffmann et al. (2018)	Under Which Conditions Are Consumers Ready to Boycott or Buycott? The Roles of Hedonism and Simplicity	Consumer Behavior	Survey	Boycott	16
Scheurenbrand et al. (2018)	Cycling into Headwinds: Analyzing Practices That Inhibit Sustainability	Lifestyle	Ethnography	Cycling	16
Yarimoglu et al. (2019)	Factors influencing Turkish parents’ intentions towards anti-consumption of junk food	Health	Survey	Food	14
Gollnhofer (2017)	The Legitimation of a Sustainable Practice Through Dialectical Adaptation in the Marketplace	Environmental Sustainability	Ethnography	General	14
Harnish and Roster (2019)	The tripartite model of aberrant purchasing: A theory to explain the maladaptive pursuit of consumption	Lifestyle	Survey	General	13
Brownlie and Hewer (2009)	Articulating consumers through practices of vernacular creativity	Lifestyle	Theoretical	General	13
Allen et al. (2018)	How knowledge, attitudes, and beliefs impact dairy anti-consumption	Health	Survey	Food	12
Lasarov et al. (2019)	Counter-arguing as barriers to environmentally motivated consumption reduction: A multi-country study	Environmental Sustainability	Secondary data	General	11

## Discussion

### Structural approach to anti-consumption

Anti-consumption manifestations seem to require an upstream social marketing analysis with inclusion of public policies, as the issues raised are above all a social issue that may not be circumscribed by downstream social marketing (Gordon, 2013). Upstream social marketing has focused on health and public health problems. Societal and environmental problems that can be related to anti-consumption can be inspired by public health, societal influences on goods and consumption, environmental and nature protection goals (Gordon, 2013). Upstream social marketing literature that merges with anti-consumption is not extensive. However, previous studies on topics like excessive drinking culture, have improved the understanding in possible paths to minimize the effects of excessive drinking culture (Fry, 2010; Piacentini & Banister, 2009). Efforts to study the phenomena highlights that extant public policies implementations require research to achieve public policy activities, like promotional and educational campaigns. It recognizes social marketing as an active tool in using marketing as an upstream activity in understanding, developing knowledge and implementing change.

The push for structural change targets the behaviors of those who contributed to the implementation, dissemination and debate of societal conditions (Gordon, 2013). Anti-consumption can boost more sustainable consumption practices such as slow consumption (Scott & Weaver, 2018) in the form of education programs or the dissemination of information to consumers, producers, designers, and those who, influenced by policy, are also beneficiaries and active participants in its development. These materializations of anti-consumption policies based on product lifecycle information are important to bridge the intention-behavior gap regarding sustainable or other social goals and the orientation of companies and producers towards innovation and the product chain. While such orientation promotes new market opportunities, brands develop their activity on this anti-consumption cultural and environmental platform, assuming informational and normative interaction with consumers. In this sense, policymaking must take into account role of brands as moderators of consumption and product life-cycle, especially in the case of slow and fast consumption cycles (Anderson et al., 2018; Evers et al., 2018).

The overriding consideration is how social marketing can effectively access those in power – who are the most contributing stakeholders (Domegan et al., 2019) –, and what are the expected theoretical, ethical, and operational challenges faced– in order to create and determine a desirable path for society at large, especially with regard to the goal of sustainable consumption and production (Gordon, 2013). Creating change requires the participation of all actors in co-creating a solution to social problems (Domegan et al., 2016). The upstream approach addresses those in power, such as companies and politics, to associate themselves with the need to change behaviors, not only regarding consumption, but also the way in which the regulation of their relationships is necessary to achieve behavioral change outcomes that are effective and desirable (Hall, 2016).

Additionally, the analysis of how stakeholders engage, clash, and collaborate is necessary to assess the contribution of marketing. The positive outcomes displayed in social marketing initiatives where the participation of communities and other audiences highlights the importance of transparency in policymaking and community involvement in this process, since a top-down approach can produce undesired outcomes (Domegan et al., 2019; Gordon, 2013; Mehmet & Simmons, 2019). Policymaking initiatives are often seen as unidirectional decisions (which can be inclusive in terms of their development), and taxation, incentives, or other types of regulation directly or indirectly impact certain agendas according to the level of influence of each stakeholder. At the same time, research and policymaking would benefit from closer ties between researchers and other stakeholders, both in acquiring and disseminating information (Hall, 2016). The target of the social problem is one of the key issues studied by social marketing, and the challenge of stakeholder interaction can harm their performance. In fact, the holistic perspective and understanding of the root causes of a given social problem seem to be one of the underlying reasons for the existence of cases of ineffective implementation (Wymer, 2021). However, specific functions regarding consumer choice apply to the relationship of consumers with public policy dissemination, and are highly dependent on products, industries, and different cultures. This growing interest in the implications of relationship between policymaking and anti-consumption is still very much open to further developments on different contextual and analytical levels (Scheurenbrand et al., 2018).

### Policymaking and systemic change

The impact of governance and dissemination activities on sustainable goals is scarcely measured and adaptation to new contexts entails risks – an issue that affects policymaking and research relationships (Isenhour, 2010). The way consumers respond to public policies is diverse and has been researched within the scope of sustainable implications (Anderson et al., 2018; Chatzidakis & Shaw, 2018).

For the systems-thinking theory, the macro approach requires the right systematic targets to achieve a cohesive model (Domegan et al., 2016). Systemic thinking applied to social marketing is a highly demanding task, both in extension and duration, that develops the knowledge and interaction between all actors and their relationships. The inclusion of stakeholders, including policymakers and community members, is an important aspect to obtain informational data that can provide a systemic change in the thinking of social marketing systems.

One of the social issues that requires change addresses environmental sustainability and the ecological problem – such as waste reduction –; this issue has been explored in the anti-consumption literature and calls for future research (Black & Cherrier, 2010; Chatzidakis et al., 2012; Dholakia et al., 2018; García-de-Frutos et al., 2018; Seegebarth et al., 2016). As current consumption is unsustainable, markets are pressured to allocate resources more efficiently, while also eliminating negative externalities in a transformational process of the whole consumer society (Holt, 2012). Anti-consumption and policymaking are not limited by cost-effective (or cost-efficient) measures, seeking effectiveness in achieving societal goals.

Anti-consumption counterintuitively expands market share as new developments at the marketing level work, and incumbents pursue strategies that not only enhance success and economic viability, but should also allow for underlying sustainable effects to occur. Innovation at the marketing level is not always transparent, and despite this sustainable orientation, its consequences and level of influence need to be measured, which is why systemic change and deeper social disruption are needed to achieve environmental sustainability goals (Isenhour, 2010; Sekhon & Armstrong Soule, 2020).

Leadership often relies on companies and public policies to raise awareness, whether through corporate social responsibility or political responsibility initiatives (Gossen et al., 2019; Isenhour, 2010; Ziesemer et al., 2019): The process resulting from public policies implementation is one of the observable sums of the interaction between the public, market actors or governments regarding anti-consumption (Hartl et al., 2016; McArthur, 2015) in health contexts (Piacentini & Banister, 2009) or policy implications in heterogeneous of groups (Cherrier & Hill, 2018). Specifically, researchers have considered how the management and development of public infrastructures can support policies that aim to improve environmental sustainability, promote reduction of consumption (Ziesemer et al., 2021), and sustainable anti-consumption practices, especially those based on substitution practices or sustainable consumption habits (Ganassali & Matysiewicz, 2021; Lee et al., 2018). Individual awareness and knowledge of the problem is a part of behavior change; how different actors participate in the discussion, their relationship with regulation, local and global operationalization is another very relevant research topic in the environmental and anti-consumption literature (García-de-Frutos et al., 2018). In this distinction, upstream social marketing campaigns can be instrumental in fostering open collaboration for the development of public policies, since community inclusion is critically important for achieving effectiveness, supporting the decision-making of its members (Issock et al., 2020; Mehmet & Simmons, 2019). Policymaking can support the communal decision-making process of each individual.

In order to reduce environmental impact, it is necessary to further investigate the phenomenon of anti-consumption in relation to the contribution of brands in improving consumption practices (García-de-Frutos et al., 2018; Sekhon & Armstrong Soule, 2020; Sussan et al., 2012). If this effect is considered insufficient considering the environmental sustainability goals, it is still a positive factor to expand sustainable practices and discussions (Reich & Soule, 2016). Other actions may be needed to solve social issues, and policy interaction with brands, communities and a given social issue requires attention from researchers and policymakers to further improve it.

### The relationship of marketing with anti-consumption

The role of the marketing mix as a structured foundation for companies in their sustainable endeavors is an important debate. Product durability, including its production methods, sourcing, material design, and repairability, are connoted with sustainability (Gossen et al., 2019). Some researchers consider a purposeful adaptation of the marketing mix to social marketing, an operational approach to promote change in society (da Silva et al., 2018; Gordon, 2012; Hall, 2016) and uncover new applications and changes to the commercial orientation (Levit & Cismaru, 2020; Wymer, 2021). Others critically analyze the role of marketing and marketing systems in society. Historically, marketing has been critically deconstructed on issues of socioeconomic inequality. This critical view of marketing serves the propose to try to extend it beyond its market functions and be based on its relationship with society (Gordon, 2011). The implications arising from marketing mix tools are not only considered regarding consumption, but also regarding anti-consumption while being promoted or applied in the marketing mix elements. The active search for alternatives to consumption, even if it is not by the final consumer – considering the effect of consumption on wellbeing, social good and environmental sustainability –, supports a marketing relationship with anti-consumption, both from a commercial and social perspective. Innovations and claims infused into marketing mix strategies combine demand and social oriented marketing mix optimizations.

Social marketing not only addresses how it can be applied for social good, but also uses critical analysis as an active process to tackle social issues to enhance the social aspect, and as such should be called upon to prevent social issues and promote environmental sustainability (Gordon, 2011). There are some generic distinctions between critical social marketing, which deconstructs the impact of marketing in society, social marketing. Therefore, there are causes to which marketing contributes, for example, socially responsible marketing, focuses on using marketing to benefit society at large, while regulating marketing activities that are harmful to society. Despite this, some consider that the regulatory aspects already fits within marketing functions.

The relationship between commercial marketing and anti-consumption takes on different forms as the demand for sustainable, environmentally, and socially responsible offers grows. This makes use of anti-consumption practices to promote consumption, whether by informing, educating or simply by using marketing to promote benefits in a sort of carrotmobbing operationalization. Exemplified in carbon labels, these marketing mix strategies can influence prices, increase or bundle sustainability-friendly offers, spawn criticism over equity, social justice and long-term effects of these commercial strategies (Gossen et al., 2019). Price premiums can pose risks that challenge social justice and equity, and can also limit resource allocation to pricing strategies and innovations, thus limiting the impact and undermining more effective options. "From a macromarketing perspective, commercial marketing techniques can only be as substantial for sufficiency as the underlying business model" (Gossen et al., 2019, p. 4). Placement can also imply anti-consumption endeavors of competing goods, especially when presenting sustainable alternatives that directly impact the environment.

Community interactions between consumers and producers challenge existing production systems as they create sustainable alternatives that rely on new distribution networks of information and goods, supported by technological innovations. Self-consumption and prosumption of solar energy at the level of final regulatory forms in Europe, allows the promotion of environmental sustainability in with the face of the existing consumption model (Gossen et al., 2019). Leaving aside niche examples of anti-consumption such as voluntary simplicity and centering the discussion on a broader, common consumer perspective allows for subtle forms of anti-consumption that are inherited by the consumption system, impacted both by social marketing and individual interaction, in constructing an impactful change (Albinsson et al., 2010; Fernandez et al., 2011; Kuanr et al., 2020).

Some conscientious, pro-environment consumers do not engage in anti-consumption. Instead of limiting themselves to the inconvenience of not consuming or boycotting based on environmental reasons, some consumers engage in carrotmobbing, promoting the acquisition of products that challenge current practices and met environmental, social, and economic criteria (Hutter & Hoffmann, 2013). Environmental measures to curb emissions, as in the case of travel and tourism, are considered too difficult to combat, but these efforts can benefit from anti-consumption (Culiberg et al., 2022; Hall, 2016). Upstream behavior change must take into consideration holistic effectiveness of incentives, and policymaking should be aware of commercial marketing activities, as well as consumer activities regarding anti-consumption and sustainability-oriented options. Environmental policymaking has received great attention in sectors such as tourism and travel, where there is environmental regulation in place, but where the effectiveness of current strategies is still under study, especially when considering an global scenario (Hall, 2016).

## Conclusion

This paper’s original and valuable contribution to the extant research starts with the link between anti-consumption consequences and policymaking. Despite promising approaches and growing interest, this connection was still under-researched. Furthermore, the scope targeted by macro studies requires a holistic perspective to increase effectiveness. This perspective on anti-consumption, especially regarding policymaking, benefits from the lenses of social marketing and macromarketing. Policymaking and the different stakeholders’ contribution have been the object of upstream social marketing research. However, based on prior knowledge on social marketing, the macro perspective remains a challenge for researchers and stakeholders in addressing complex social issues. The way policymaking interacts with marketing, citizens, and other actors on the local and global stage at the same time needs to be accounted for and understood, as well as allow for a contextual focus to be effective. The deconstruction of marketing by social marketing can contribute to the duality in application of marketing both as a framework for researching anti-consumption and as an object of operationalization of anti-consumption.

This research shows how anti-consumption is linked, via mechanisms of environmental sustainability, to specific issues of social marketing that are studied in the literature and are especially patent in the UN’s Sustainable Development Goals, which include, among others, Sustainable Consumption and Production Practices. Within the social marketing paradigm, anti-consumption appears as upstream and downstream applications with examples of behavior change at the micro, meso, and macro levels that link policy to a driver of change.

Stakeholder theories are important for the conceptual development of anti-consumption, as policymakers and the scope of policies are mechanisms at the levels of consumption, production, and distribution, where the approach of the participants and the level of importance determine and are determined by a system where anti-consumption practices take place. Finally, critical social marketing adds the opportunity to look at social issues on anti-consumption through marketing operations.

In sum, the current systematic literature review uncovered different logical steps to further research on anti-consumption towards a macro approach. As social marketing proposes, the number of different stakeholders needs to be mapped to offer a model the operation of anti-consumption mechanisms through public policy. Additionally, it contributes to understanding the impact of anti-consumption on policymaking, and scope of challenges of sustainable societal goals, including geopolitical, in achieving global alignment.

## Limitations and future research

Multi-level and multinational scope approach is of great research interest, as it offers interdisciplinary opportunities, but it also highlights the problem of finding global strategies that are locally effective. One of the limitations observed by some macro approaches, especially in social marketing, is to provide an international framework of work that can be truly global, as supranational organizations try to find cooperation between nations. Looking at the systemic roots of anti-consumption, building—through the consumers within their marketing system—a broader set of issues and causes that can respond to macro and multinational challenges, such as the United Nations World Tourism Organization (UNTWO), can provide an effective shift to a sustainable level in tourism. Although the contribution to conceptual and theoretical knowledge is important, the empirical testing of this macro research has important challenges: longitudinal, timeframe, and complexity.

Applications in the literature consider anti-consumption and sustainable practices of great interest to managerial implications. Product lifecycle labels and marketing mix are prime examples. Brand signaling is important for brands to support environmental sustainability, but their effects on the marketing mix, and the interaction of the consumer with these tools, calls for further research and its macro applicability to change consumption patterns. Further research needs to account for policymaking and stakeholders, for applications of anti-consumption in macromarketing, considering issues like social injustice and inefficiency. It is also important to consider marketing mix strategies that influence branding, pricing, but also the role and relationship of innovation along the value chain. In recent years, solar energy systems are one of the examples where policy has introduced promising changes in the market, where cooperation capabilities move away from the traditional electrical grid system, also connected to reduction and essential policy interaction between nations providing examples of macro applications. The interaction of policy with travel and tourism environmental sustainability, call for solution in the upstream social level to seek sustainable effective strategies. There are opportunities for a structural and fundamental change in pursuing sustainable goals considering anti-consumption, and many macro applications need further consideration from countries and industries.
